# Abdominal wall endometriosis: a case report

**DOI:** 10.11604/pamj.2022.41.193.33536

**Published:** 2022-03-10

**Authors:** Daniel Paramythiotis, Eleni Karlafti, Ioannis Tsomidis, George Iraklis, Petra Malliou, Anestis Karakatsanis, Michalopoulos Antonios

**Affiliations:** 1 First Propaedeutic Surgery Department, AHEPA University General Hospital of Thessaloniki, 54636 Thessaloniki, Greece,; 2 Emergency Department, AHEPA University General Hospital of Thessaloniki, 54636 Thessaloniki, Greece,; 3 First Propaedeutic Internal Medicine Department, AHEPA University General Hospital of Thessaloniki, 54636 Thessaloniki, Greece

**Keywords:** Endometriosis, endometrioma, rectus abdominus, abdominal wall mass, case report

## Abstract

Abdominal wall endometriosis is the development of endometrial tissue in the anterior abdomen usually due to an operation in which the uterus is manipulated. We herein delineate the presentation, clinical investigation, and surgical treatment of an abdominal wall endometriosis case. A 42-year-old female presented with acute abdominal pain in the lower quadrants in the margins of an old cesarean scar. Two masses in the abdominal wall highly suspected of consisting of endometrial tissue were found during the investigation of the patient. These ones were removed in surgery and endometrial tissue secondary to previous cesarean section was confirmed after histological analysis. Consequently, although rare, if a painful mass in a surgical scar, such as a Pfannenstiel incision, is found in women of reproductive age with a history of obstetric surgery, the differential diagnosis shall include endometriosis. There is a portion of cases in which endometriosis recurs within five years following conservative surgery.

## Introduction

Endometriosis is defined as the presence of ectopic endometrial tissue that can respond to ovarian hormonal stimulation [1]. Endometriosis´ typical characteristics include chronic infertility and pain and its incidence is estimated around 10% of women of reproductive age [2]. In most cases, endometriosis is located within the pelvis. Nevertheless, ectopic endometrial tissue can be found outside the pelvis and can affect different organs, causing a variety of symptoms, occasionally with cyclical symptoms [3]. The bowel, the urinary system (kidney, ureter, bladder), lymph nodes, and the abdominal wall are the main sites targeted by extra pelvic endometriosis [4,5]. Endometriosis is also seen in perineum after vaginal deliveries with episiotomy [4]. There are a few cases of endometriosis after appendectomy, inguinal herniorrhaphy, in the laparoscopic trocar port site, and amniocentesis needle tract. The most common site of extra pelvic endometriosis is the abdominal wall (4%) [6] and is associated with prior surgical scars following gynecologic abdominal procedures like caesarean section, hysterotomy, hysterectomy, tuballigation or myomectomy [2]. Diagnosis is made preoperatively with non-pathognomonic imaging techniques like Ultrasound Sonography (USG), Computed Tomography (CT) scan, Magnetic Resonance Imaging (MRI) scan, and Fine Needle Aspiration Cytology (FNAC) or after wide surgical excision followed by histopathologic examination of the excised tissue [7]. Abscess, suture granuloma, hematoma, desmoid tumor, sarcoma and metastatic malignancy are entities that should be included in the differential diagnosis of abdominal wall endometriosis [8-10]. We present a case of abdominal wall endometriosis, which developed in the scar of caesarean section.

## Patient and observation

**Patient information:** a 42-year-old female presented at the surgical emergency department complaining of a painful swelling in her lower abdomen. The pain in the swelling was a dull ache which would aggravate during menstruation. The patient´s medical history included two cesarean procedures via a pfannenstiel incision.

**Clinical findings:** the vital signs were normal and she was afebrile. During clinical examination, the palpation of the abdomen revealed two lumps. The one lump was in the right lower quadrant and the other one in the left lower quadrant, while both of them were near the midline.

**Figure 1:**
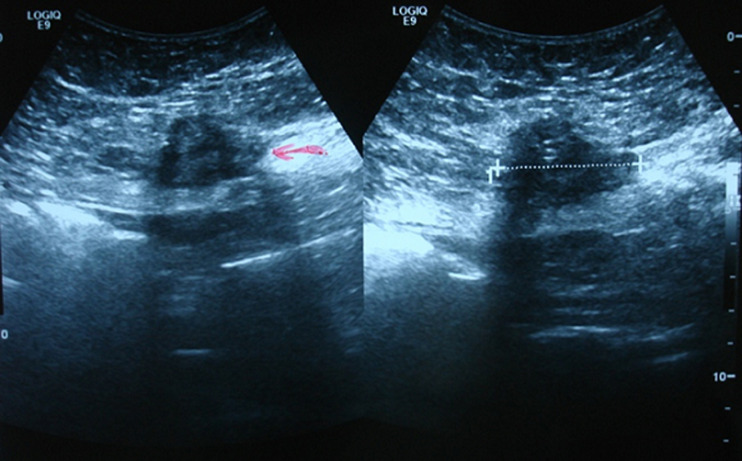
ultrasound sonography (arrow), 42mm round mass in the anterior abdominal wall

**Figure 2:**
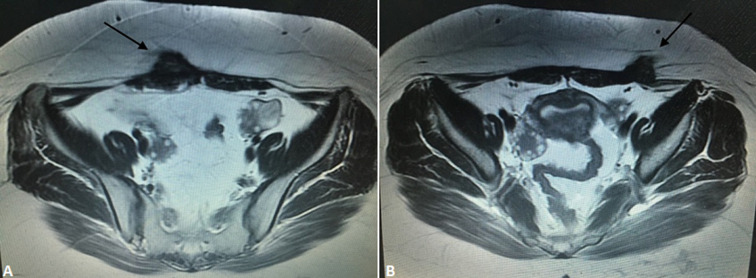
(A, B) MRI images of the masses in the anterior abdominal wall (arrows); the lesions tend to extent to the subcutaneous fat and the right rectus abdominis muscle

**Diagnostic assessment:** the abdominal USG showed a 42mm round, solid mass, with poor blood flow in the anterior abdominal wall (Figure 1). She also underwent an MRI scan, which showed two solid masses with ill-defined margins, one on the right side of the midline, with 3.5cm diameter, and the second one on the left side, with 2.5cm diameter. The two lesions demonstrated with low signal on T1 images, high inhomogeneous signals on T2-weighted images and pathological contrast enhancement using intravenous paramagnetic contrast media. The lesions tend to extent to the subcutaneous fat and the right rectus abdominis muscle (Figure 2 (A, B)).

**Figure 3:**
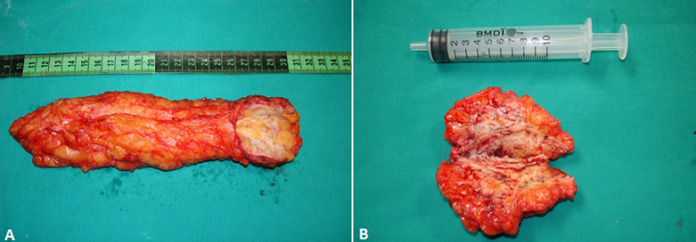
(A, B) the removed mass during surgery

**Figure 4:**
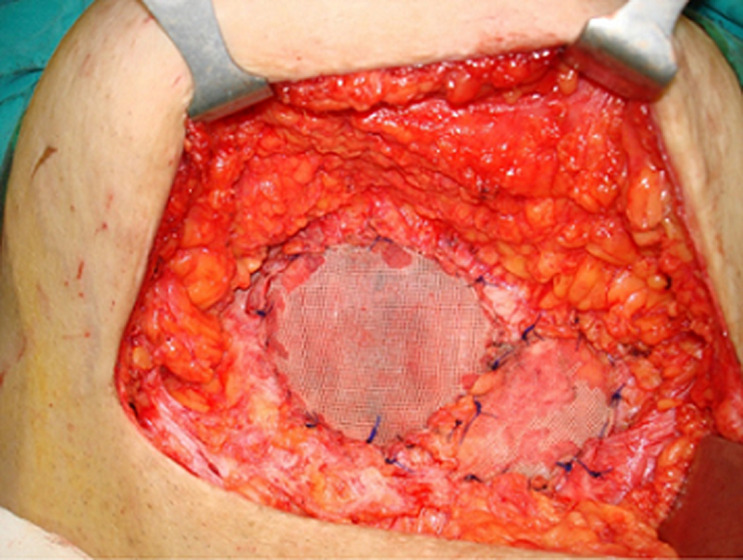
abdominal wall reconstruction by using prolene mesh

**Therapeutic interventions:** the patient, under general anesthesia, underwent wide local resection of the masses. In the operating room, the mass and the local abdominal wall removed en bloc, because endometrial tissue was developed at the rectus abdominus muscle sheath too (Figure 3 (A, B)). The abdominal wall was reconstructed by using prolene mesh (Figure 4).

**Diagnosis:** the histologic examination evinced the presence of endometrioma -more specific: multiple endometrial glands with variable focal cystic dilatation, surrounding specialized stroma and dense fibrosis between the endometriotic foci. There were no signs of cellular atypia in the histopathological examination of the tissue.

**Follow-up and outcome of interventions:** there were no complications postoperatively and the patient discharged five days later. The woman was disease- free for endometriosis after 1 year at her scheduled follow up appointment.

**Patient perspective:** the patient was satisfied with the diagnostic and the proposed care.

**Informed consent:** the patient gave permission for her images and other clinical information to be reported in the journal. It has been known to the patient that her name and initials will not be published.

## Discussion

Endometriosis is the presence of functioning endometrial tissue outside the uterine cavity. Endometriosis of the abdominal wall is a rare entity and difficult to diagnose as the symptoms may be non-specific [1]. The clinical diagnosis of rectus abdominis muscle mass lesions could be hernia, lipoma, hematoma, abscess, and benign as well as malignant tumor [9]. The pathophysiology of endometriosis remains vague. According to Sampson´s theory of implantation [9] which is the most accepted, the reflux of endometrial fragments regurgitated through the fallopian tubes during menstruation results in the subsequent endometrial tissue implantation on the peritoneum and the ovary [10]. Aberrant migration or differentiation of the Muüllerian ducts can be the reason of the development of endometrial tissue [11]. Ovarian endometriosis can be explained by the theory of coelomic metaplasia [12,13].

The average age at presentation is 31 years [8]. The incidence of the disease is between 6% and 10% of all women and 35-50% of women with infertility and pelvic pain. Abdominal wall endometriosis is a rare condition that can develop after open uterine surgeries [12]. During caesarean section, the needle passing through the endometrium may inoculate endometrial tissue in the abdominal wall when it is being stitched with the same needle [9,12]. Endometriosis should be considered as a chronic inflammatory disease because the ectopic tissue is associated with overproduction of prostaglandins, cytokines and chemokines [14]. It is highly indicated that estrogen has not only proliferative but also proinflammatory and antiapoptotic role in the cells of the endometrial tissue [5,14]. These effects seem to be magnified in women with endometriosis, in whom local estradiol reinforces both inflammation and cell survival. The symptoms do not have always-cyclic attribute and the imaging procedures are not specific for the diagnosis [14]. The real incidence of scar endometriosis is estimated at 0.03% to 0.15% with the first symptoms starting around five years after the procedure has launched [12]. The main clinical manifestations of endometriosis are pain, cyclic or not, and infertility. Endometriosis- associated infertility, which affects up to 30 to 50% of patients [14], can occur due to loss of ovarian function, abnormalities of the eutopic endometrium, alteration of the fertilization process, pelvic adhesions, dyspareunia and possible surgical damage to the ovary [13,14].

Risk factors for endometriosis include early menarche, shorter than 27-day menstrual cycles, small number of childbirths, age 25-29, Caucasian race, daily consumption of ample amount of alcohol, excessive red meat consumption, and smoking [1,14]. The USG is the optimal choice for the diagnosis of the disease. The mass is characterized as hypoechoic and heterogeneous with scattered internal echoes [2,4,5]. In some cases, the masses appear totally solid but occasionally some cystic changes may be seen [5]. Magnetic resonance imaging outweighs USG because of its ability to detect masses that imitate endometriosis on the abdominal wall and should be regarded as the second-line imaging technique [1]. Computed tomography findings depend on the phase of the menstrual cycle [15]. Masses might appear mostly solid, cystic, or as a mixed appearance of both elements [15]. Because endometriotic lesions can present as a mass lesion, it has been proposed to investigate them by FNAC [9], often under USG or computed tomographic guidance [11].

Medical treatment with the use of oral contraceptive pills, progestogens, and danazol is not efficacious to cure the patient and gives only incomplete relief in symptoms [8] and, simultaneously, it can bring about several adverse effects [1]. The clinical improvement using hormonal treatment observed for endometriotic implants at other sites has not been observed for abdominal wall endometriosis [2]. Treatment of choice for abdominal wall endometriosis is considered to be wide surgical excision with at least a 1cm margin with patch grafting of the defect [8].

Good surgical techniques and proper care during cesarean section might prevent scar endometriosis [16]. It has been suggested that at the end of surgery (especially manipulations of uterus and tubes), thorough cleaning with high jet saline solution before closure could decrease the relative risk of developing endometriosis [17]. There is high possibility of recurrence of endometriosis, subsequently follow up in patients with this disorder is needed [2]. Additionally, possibility of malignancy should be ruled out in cases of continual recurrence [18]. First-degree relatives of patients with endometriosis are up to 6 times more likely to develop endometriosis. Furthermore, according to large studies of twins heritability is approximately 50%. Although heritability is approximately 50% according to studies of twins, the genetic background of the disease remains unclear [19].

## Conclusion

Scar endometriosis is a rare entity and often elusive that can result to frustration. A high level of suspicion should be maintained in any woman presenting with pain and swelling at an incisional site. History and physical examination are always helpful and every surgeon should consider endometriosis in their differential diagnosis. Wide excision is the treatment of choice whereas follow-up is required due to the possibility of recurrence.
